# Clinical Characteristics of Geriatric Patients With *de novo* Parkinson’s Disease Compared with the Non-Geriatric Population: Adapting to Changes in the Era of Aging

**DOI:** 10.31083/RN39115

**Published:** 2026-01-21

**Authors:** Kyum-Yil Kwon, Jihwan You, Rae On Kim

**Affiliations:** ^1^Department of Neurology, Soonchunhyang University Seoul Hospital, Soonchunhyang University School of Medicine, 04401 Seoul, Republic of Korea

**Keywords:** aged, cognition, geriatrics, Parkinson’s disease

## Abstract

**Background:**

Parkinson’s disease (PD) is increasingly being diagnosed in older adults. Despite this trend, the clinical features of geriatric patients with PD are not thoroughly defined. This study aimed to compare the clinical characteristics of geriatric patients (aged ≥75 years) with *de novo* PD against those of non-geriatric patients (aged <75 years) newly diagnosed with PD.

**Methods:**

This retrospective analysis enrolled 110 patients aged 50 years or older with *de novo* PD from our hospital’s Parkinsonism registry between 2017 and 2023. Clinical evaluations included motor assessment via the Unified Parkinson’s Disease Rating Scale Part III and global cognitive function was measured using the Montreal Cognitive Assessment (MoCA). Nonmotor symptoms, including depression, anxiety, and fatigue, were assessed using other scales and autonomic dysfunction was assessed using the Scale for Outcomes in Parkinson’s Disease–Autonomic (SCOPA-AUT).

**Results:**

Geriatric patients with PD (n = 37) exhibited significantly lower cognitive performance (lower MoCA scores, *p* < 0.001) and more pronounced autonomic dysfunction (higher SCOPA-AUT scores, *p* = 0.0103) in comparison with non-geriatric PD patients (n = 73). In multivariate logistic regression analysis, lower MoCA scores (odds ratio [OR]: 0.7642, 95% confidence interval [CI]: 0.6712–0.8701, *p* < 0.001) and elevated SCOPA-AUT scores (OR: 1.0640, 95% CI: 1.0031–1.1286, *p* = 0.0391) emerged as significant independent predictors of geriatric PD.

**Conclusions:**

These findings reveal a distinct clinical phenotype among geriatric patients with *de novo* PD, underscoring the value of early detection and proactive management of cognitive and autonomic impairments in this group. The results further emphasize the need for individualized assessment and therapeutic interventions tailored to the specific requirements of geriatric patients with PD.

## 1. Introduction

Parkinson’s disease (PD) is showing growing prevalence among older adults, especially those of advanced age. There is an increasing influx of geriatric individuals with PD seeking care at movement disorders clinics [1]. This trend has been referred to as a global “Parkinson pandemic”, primarily attributed to population aging and changing demographics [2]. Furthermore, recent investigations have documented a substantial rise in the global burden of PD in recent decades [3, 4]. Collectively, these observations highlight an urgent need for targeted approaches to optimize the management of geriatric PD in aging societies.

In many countries, individuals aged 60 or 65 years and older are conventionally designated as “elderly” or “geriatric people”, based on chronological age. Nonetheless, the rapid expansion of the aging population during the 21st century has led several nations to become aging societies. In recent years, super-aged societies such as Japan have initiated a redefinition of the “geriatric” threshold, shifting it from 65 to 75 years [5]. This change recognizes both increased life expectancy and the enhanced health status of older adults, thereby encouraging a reconsideration of population classifications and tailored approaches to their care requirements. Furthermore, clinical research increasingly employs 75 years as the age cutoff for older patient groups, ensuring that study designs are better aligned with evolving demographic trends [6, 7, 8].

Despite these demographic changes, the clinical characteristics of newly diagnosed (*de novo*) PD in people aged 75 years and older have not been thoroughly explored. Our objective was to examine the clinical features of geriatric PD patients aged 75 and above, in comparison with non-geriatric PD patients. The findings of this study are intended to enhance our knowledge of PD manifestation in the geriatric cohort and provide important perspectives for the development of more personalized clinical management strategies.

## 2. Materials & Methods

### 2.1 Patients

**Fig. 1.**
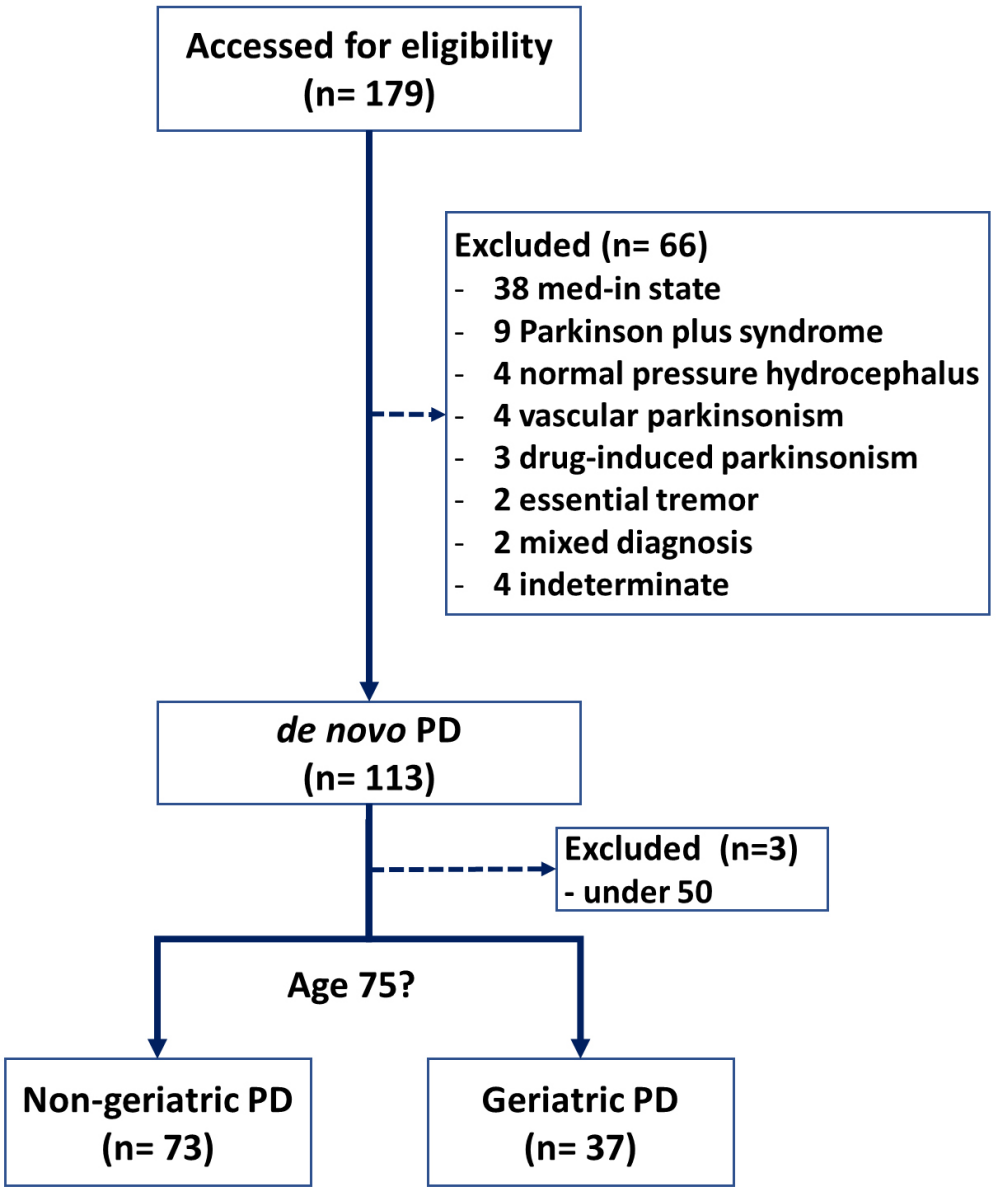
**Flowchart of the study**. PD, Parkinson’s disease.

The Institutional Review Board of our hospital approved this retrospective study and granted a waiver of informed consent (approval number: 2025-01-003). Between 2017 and 2023, 179 patients were enrolled in our Parkinsonism registry. We included only *de novo* PD patients who had a follow-up period exceeding one year. At our movement disorders clinic, the diagnosis of PD was established according to the UK Parkinson’s Disease Society Brain Bank criteria [9]. Additionally, brain magnetic resonance imaging (MRI) and 18F-fluoropropyl-carbomethoxy-3β-(4-iodophenyl)-N-(3-fluoropropyl) nortropane positron emission tomography (18F-FP-CIT PET) were employed to distinguish between Parkinson plus syndrome and secondary parkinsonism, accompanied by clinical follow-up for a minimum of one year [10, 11]. Thirty-eight patients were excluded due to not being drug-naive at baseline. Fifteen patients were classified as having atypical parkinsonism, including multiple system atrophy (n = 5), progressive supranuclear palsy (n = 3), essential tremor (n = 2), dementia with Lewy bodies (n = 1), and unspecified parkinsonism (n = 4). Thirteen patients had secondary parkinsonism, comprising drug-induced parkinsonism (n = 3), normal pressure hydrocephalus (NPH, n = 4), vascular parkinsonism (VP, n = 4), and a possible dual diagnosis of NPH and VP (n = 2). Patients with PD under 50 years old were further excluded, given that their clinical characteristics may differ from those typically seen in older PD patients with classic disease features [12, 13]. Consequently, three patients were removed from the study because they were younger than 50 years at registration. This led to the inclusion of 110 patients with *de novo* PD in the final analysis (Fig. 1). Patients were categorized based on registration age: those 75 years or older were designated as geriatric PD (n = 37), and those younger than 75 were designated as non-geriatric PD (n = 73).

### 2.2 Clinical Assessments

All patients received thorough clinical assessments at the time of enrollment. Demographic and clinical information such as age, gender, body weight, height, and educational attainment was collected. Motor symptoms were evaluated with the Unified Parkinson’s Disease Rating Scale Part III (UPDRS-III), and disease severity was determined according to the Hoehn and Yahr (H&Y) staging [14]. Global cognitive performance was measured using the Korean version of the Montreal Cognitive Assessment (MoCA) [15], and depressive symptoms were assessed with the Beck Depression Inventory (BDI) [16]. Anxiety was evaluated with the Beck Anxiety Inventory (BAI) [17], and fatigue levels were measured using the Parkinson’s Disease Fatigue Scale (PFS) [18]. Furthermore, autonomic function was assessed using the Korean version of the Scales for Outcomes in Parkinson’s Disease – Autonomic (SCOPA-AUT) [19], with the sexual dysfunction domain omitted due to incomplete patient responses. The clinical data were examined to distinguish geriatric and non-geriatric Parkinson’s disease groups in terms of motor, cognitive, psychological, and autonomic domains.

### 2.3 Statistics

Statistical evaluations were performed to contrast clinical characteristics between the non-geriatric and geriatric PD groups. Continuous data are reported as mean ± standard deviation (SD) for Student’s *t*-test or medians with interquartile ranges (IQR) for the Mann–Whitney *U* test, after testing for normality using the Kolmogorov-Smirnov test. Categorical data were expressed as number (percentage) and compared utilizing the χ^2^ test or Fisher exact test as suitable. To determine factors independently associated with geriatric Parkinson’s disease, multivariable logistic regression analyses with stepwise selection were used. Variables reaching a *p*-value < 0.2 during univariable analysis were incorporated into the multivariable model. Odds ratios (ORs) and 95% confidence intervals (CIs) were derived for all logistic regression analyses. A threshold of *p*-value < 0.05 indicated statistical significance. Rex software version 3.6.3 (RexSoft Inc., Seoul, South Korea) was employed for all statistical analyses.

## 3. Results

### 3.1 Comparison of Clinical Features Between Non-Geriatric and Geriatric PD

**Fig. 2.**
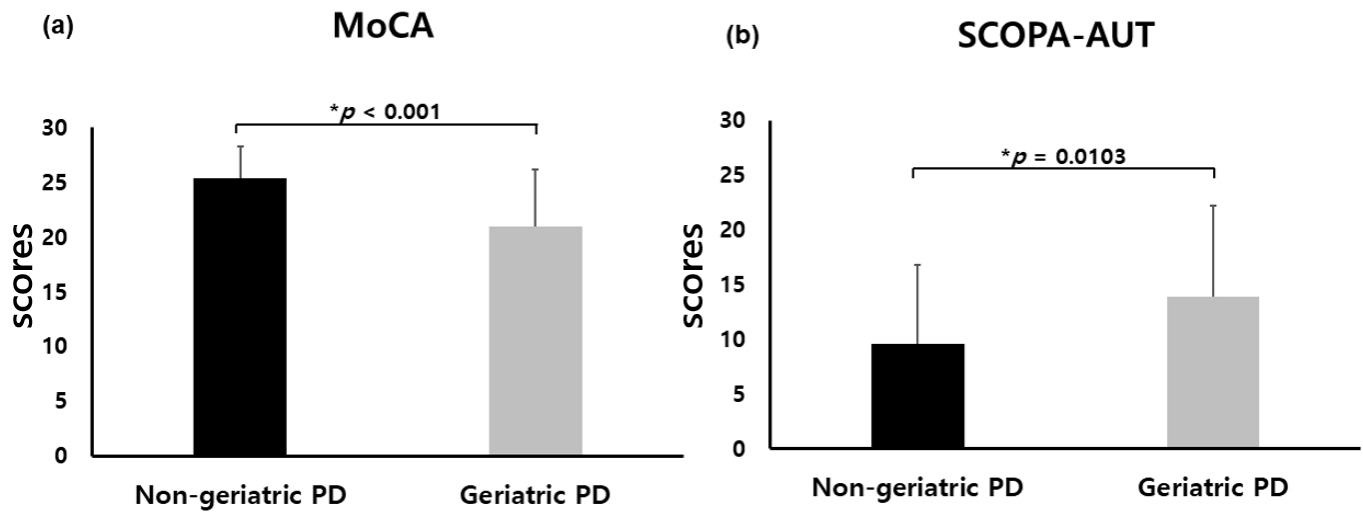
**Clinical distinctions between geriatric and non-geriatric patients diagnosed with *de novo* Parkinson’s disease (PD)**. Geriatric patients with *de novo* PD exhibited lower MoCA scores (a) and elevated SCOPA-AUT scores (b) relative to their non-geriatric counterparts.

Geriatric individuals (age ≥75 years) with *de novo* PD showed notably different clinical characteristics in comparison to non-geriatric individuals (age <75 years) (Table 1). Geriatric individuals were older (79.49 ± 3.13 years vs. 66.18 ± 5.98 years, *p* < 0.001) and had lower average height (1.58 ± 0.09 m vs. 1.62 ± 0.08 m, *p* = 0.0265). They also had fewer years of formal education (7.88 ± 4.88 years vs. 11.29 ± 4.70 years, *p* < 0.001) and demonstrated reduced cognitive ability as evidenced by lower MoCA scores (20.95 ± 5.17 vs. 25.32 ± 2.95, *p* < 0.001) (Fig. 2a). Moreover, these patients obtained higher SCOPA-AUT scores, which reflect greater severity of autonomic dysfunction (13.86 ± 8.32 vs. 9.62 ± 7.21, *p* = 0.0103) (Fig. 2b).

**Table 1.** **Comparison of clinical features between non-geriatric and geriatric patients with *de novo* Parkinson’s disease**.

Variable	Total (n = 110)	Non-geriatric PD (n = 73)	Geriatric PD (n = 37)	*p* value
Age, yr	70.65 ± 8.17	66.18 ± 5.98	79.49 ± 3.13	<0.001
Female gender	57 (51.82%)	36 (49.32%)	21 (56.76%)	0.5919
Body weight, kg	60.47 ± 10.16	61.57 ± 9.86	58.30 ± 10.54	0.1206
Height, m	1.60 ± 0.09	1.62 ± 0.08	1.58 ± 0.09	0.0265
Body mass index	23.37 ± 2.69	23.41 ± 2.71	23.28 ± 2.69	0.8125
Disease duration, yr	1.29 ± 0.93	1.35 ± 0.96	1.17 ± 0.86	0.3093
Years of education	10.14 ± 5.01	11.29 ± 4.70	7.88 ± 4.88	<0.001
Diabetes mellitus, n (%)	29 (26.36%)	19 (26.03%)	10 (27.03%)	>0.99
Hypertension, n (%)	55 (50%)	35 (47.95%)	20 (54.05%)	0.6865
History of falls, n (%)	44 (40%)	27 (36.99%)	17 (45.95%)	0.4837
UPDRS-III (motor)	22.29 ± 11	21.44 ± 10.75	23.97 ± 11.45	0.2668
H&Y stage	2 (2, 2)	2 (2, 2)	2 (2, 2)	0.7705
MoCA-K (cognitive assessment)	23.85 ± 4.35	25.32 ± 2.95	20.95 ± 5.17	<0.001
BDI (depressive symptoms)	8.16 ± 6.97	7.49 ± 7.03	9.49 ± 6.74	0.1529
BAI (anxiety assessment)	5.41 ± 6.01	5.11 ± 5.92	6.00 ± 6.22	0.4734
PFS (fatigue assessment)	39.47 ± 16.81	37.81 ± 17.59	42.76 ± 14.83	0.1246
SCOPA-AUT (dysautonomia)^#^	11.05 ± 7.82	9.62 ± 7.21	13.86 ± 8.32	0.0103

This table was summarized appropriately based on the normality assessment using the Kolmogorov-Smirnov test and the presence of a chi-squared test warning: numerical data are expressed as mean ± SD or interquartile range, while non-numerical data are reported as number (%). ^#^Total score of SCOPA-AUT was calculated excluding the sexual domain, as a substantial number of patients did not respond to the question regarding sexual dysfunction. UPDRS, Unified Parkinson’s Disease Rating Scale; H&Y, Hoehn and Yahr; MoCA-K, Korean version of the Montreal Cognitive Assessment; BDI, Beck Depression Inventory; BAI, Beck Anxiety Inventory; PFS, Parkinson’s disease Fatigue Scale; SCOPA-AUT, Scales for Outcomes in Parkinson’s disease – Autonomic.

### 3.2 Association Logistic Regression Analyses for Geriatric PD

To further identify factors characteristic of geriatric PD, we conducted logistic regression analyses as presented in Table 2. Univariable logistic regression analysis demonstrated that shorter height (OR 0.0040, 95% CI 0.0001–0.4856, *p* = 0.0242), lower educational attainment (OR 0.8622, 95% CI 0.7882–0.9433, *p* = 0.0012), reduced MoCA scores (OR 0.7547, 95% CI 0.6621–0.8603, *p* < 0.001), and higher SCOPA-AUT scores (OR 1.0726, 95% CI 1.0176–1.1306, *p* = 0.0091) were all significantly correlated with geriatric PD. Other clinical factors, such as gender, body weight, motor assessment (UPDRS-III), and psychiatric scales (BDI, BAI, and PFS), did not show significance. In multivariable logistic regression analysis with stepwise selection, only reduced MoCA scores (OR 0.7642, 95% CI 0.6712–0.8701, *p* < 0.001) and higher SCOPA-AUT scores (OR 1.0640, 95% CI 1.0031–1.1286, *p* = 0.0391) were determined as independent predictors for geriatric PD.

**Table 2.** **Logistic regression analysis of clinical features associated with geriatric PD in *de novo* PD patients**.

Variable	Univariable			Multivariable		
	Odd ratio	95% CI	*p*-value	Odd ratio	95% CI	*p*-value
Gender-female	1.3490	0.6086–2.9900	0.4611			
Body weight, kg	0.9678	0.9294–1.0077	0.1127			
Height, m	0.0040	0.0001–0.4856	0.0242			
Body mass index (kg/m^2^)	0.9820	0.8468–1.1389	0.8105			
Disease duration, yr	0.7972	0.5085–1.2500	0.3234			
Educational level, yr	0.8622	0.7882–0.9433	0.0012			
Diabetes mellitus, n (%)	1.0526	0.4304–2.5743	0.9105			
Hypertension, n (%)	1.2773	0.5780–2.8229	0.5452			
History of previous falls, n (%)	1.4481	0.6491–3.2306	0.3657			
UPDRS-III (motor)	1.0212	0.9850–1.0587	0.2541			
H&Y stage	1.0432	0.4050–2.6870	0.9302			
MoCA-K (cognitive function)	0.7547	0.6621–0.8603	<0.001	0.7642	0.6712–0.8701	<0.001
BDI (depressive symptoms)	1.0410	0.9840–1.1013	0.1620			
BAI (anxiety)	1.0244	0.9604–1.0927	0.4633			
PFS (fatigue)	1.0180	0.9938–1.0427	0.1458			
SCOPA-AUT (dysautonomia)^#^	1.0726	1.0176–1.1306	0.0091	1.0640	1.0031–1.1286	0.0391

Multivariable logistic regression with step-wise variable selection was implemented. CI, confidence interval; UPDRS-III, the Unified Parkinson’s disease rating scale-part 3. ^#^Total score of SCOPA-AUT was calculated excluding the sexual domain, as a substantial number of patients did not respond to the question regarding sexual dysfunction.

## 4. Discussion

In this study, we categorized the study population into two groups: individuals with PD younger than 75 years and geriatric individuals with PD aged 75 and above, with a particular emphasis on drug-naïve *de novo* patients. Until the early 2000s, PD was generally believed to develop primarily in individuals around the age of 60 [20]. As the incidence of PD has risen among the very elderly, research on the distinguishing features of geriatric Parkinson’s disease has gradually emerged. Recent investigations have demonstrated that individuals aged 75 and older with parkinsonism under medication exhibit unique clinical and functional profiles when compared to younger individuals, with age and disease duration playing a significant role in symptom trajectory and treatment efficacy [21, 22, 23]. Furthermore, as previously mentioned, we excluded young-onset PD patients under the age of 50, since it is well-established that these younger patients display clinical features that differ from those with onset around their 60s [12, 13]. Taken together, this represents the first study dedicated to examining the clinical features of geriatric PD. More specifically, the aim of this research was to assess differences in clinical traits between PD patients aged 75 or older and those around 60 years of age. By conducting this study, we sought to delineate critical clinical characteristics that warrant increased attention from clinicians when initially evaluating geriatric PD patients, not only in clinical practice but also within the context of research.

Geriatric PD patients were observed to be shorter in stature and to have attained a lower education level when compared with non-geriatric PD patients (Table 1). These disparities are likely attributable to the distinct socioeconomic background in Korea, heavily shaped by modernization during the 1900s and the Korean War in the 1950s. Nevertheless, there were no significant differences identified between the two groups with respect to metabolic conditions including diabetes and hypertension. Additionally, no substantial variations were detected regarding the timing of their first hospital visit for PD symptoms, the severity of motor symptoms at initial presentation, or the history of falls between the groups. These results indicate that, within a relatively stable society, geriatric PD patients are unlikely to show notable differences in demographic variables or motor symptoms when compared to non-geriatric PD patients, excepting differences related to age itself.

Interestingly, we observed that the pattern of non-motor symptoms varied between geriatric and non-geriatric patients with PD. No significant differences were found between the two groups regarding non-motor symptoms such as depression, anxiety, and fatigue. However, the geriatric group exhibited lower MoCA scores, indicating more pronounced cognitive impairment, and higher SCOPA-AUT scores, reflecting increased severity of autonomic dysfunction (Table 1 and Fig. 2). To assess whether these differences were independent of variables such as education level and age, we conducted logistic regression analysis and verified that both MoCA and SCOPA-AUT independently correlated with geriatric PD (Table 2). These results underscore the necessity for clinicians to closely monitor cognitive impairment and autonomic symptoms in the management of geriatric PD patients.

It is widely recognized that older individuals with PD experience a more rapid progression of cognitive decline and develop dementia earlier compared to younger patients [24]. Furthermore, a previous meta-analysis demonstrated that cognitive impairment in PD is associated with advanced age, lower educational levels, longer disease duration, higher levodopa dosages, greater severity of motor symptoms, as well as apathy and depression [25]. Consistent with existing literature, our findings show that cognitive impairment is a significant factor even at the very early stage of geriatric PD, regardless of other clinical characteristics (Table 2). The cognitive decline identified in geriatric PD patients highlights the importance of performing early and regular cognitive assessments in this demographic. To date, the exact pathophysiological mechanisms underlying cognitive deficits linked to aging in PD remain unclear. One plausible explanation is that more individuals in the older age group with PD have concomitant pathological alterations characteristic of Alzheimer’s disease [26]. Therefore, even in cases of *de novo* PD, cognitive decline could be more evident in this subgroup. Another potential contributing factor is that aging may disrupt the integrity of the blood-brain barrier, leading to impaired immune responses and subsequent neurodegeneration, which may facilitate cognitive deterioration in the geriatric population [27]. Furthermore, aging is associated with increased iron accumulation in the brain, particularly in deep gray matter structures such as the hippocampus and basal ganglia. This phenomenon may lead to cognitive decline, especially among geriatric patients with PD [28, 29].

Autonomic dysfunction in PD may manifest as early as the prodromal stage, with a well-documented trend of progressive deterioration as the disease advances [30]. Recent evidence indicates that patients with PD who exhibit severe autonomic symptoms tend to experience significantly poorer outcomes compared to those presenting with milder symptoms [31]. The transmission of α-synucleinopathy and resulting neurodegenerative processes from the peripheral nervous system to the central nervous system is described as the gut-brain axis, which constitutes a fundamental pathophysiological pathway in PD [32]. Importantly, our findings demonstrated that autonomic dysfunction was notably more pronounced in newly diagnosed geriatric patients with PD relative to non-geriatric counterparts. These results imply that while the progression of pathological changes within the peripheral nervous system may occur at a similar tempo in geriatric and non-geriatric PD populations, the degree of severity may be distinct. Nevertheless, since this study utilized a small sample, additional investigations employing varied research designs are warranted to confirm these observations.

Several limitations should be acknowledged for this study. First, the retrospective design conducted within a single institution introduces the risk of selection bias, potentially restricting the general applicability of our results. Second, although global cognitive function was measured using the MoCA, we did not incorporate in-depth assessments of specific cognitive domains. In addition, cognitive impairment can be confounded by subclinical cerebrovascular disease, sleep disorders, or polypharmacy. We could not address in this study. Third, the evaluation of autonomic dysfunction relied upon the SCOPA-AUT, a subjective instrument, and objective physiologic measurements were not included. Despite these constraints, our study successfully identified unique clinical features in geriatric patients newly diagnosed with PD. However, especially in geriatric people, self-reported autonomic assessments may have limitations in accuracy and reliability due to factors such as cognitive impairment or recall bias. Future studies incorporating neuroimaging (e.g., hippocampal volume, white matter hyperintensity) or biomarkers (e.g., plasma pTau, α-synuclein) are required to address these limitations and further confirm our findings.

## 5. Conclusions

In conclusion, geriatric patients with PD demonstrated lower cognitive performance and more pronounced autonomic dysfunction compared with non-geriatric patients with PD. In multivariable logistic regression analysis, decreased cognitive scores and higher autonomic dysfunction scores were identified as significant independent predictors of geriatric PD. Our data indicate that geriatric patients with *de novo* PD display more substantial cognitive deficits and greater autonomic dysfunction than non-geriatric *de novo* PD patients. These findings underscore the importance for clinicians to recognize these features when treating geriatric patients and to ensure these considerations are integrated into clinical research.

## Data Availability

The data underlying this article will be shared on reasonable request to the corresponding author.

## Abbreviations

PD, Parkinson’s disease; BMI, Body mass index; MRI, magnetic resonance imaging; 18F-FP-CIT PET, 18F-fluoropropyl-carbomethoxy-3β-(4-iodophenyl)-N-(3-fluoropropyl)nortropane positron emission tomography; NPH, normal pressure hydrocephalus; VP, vascular parkinsonism; UPDRS-III, Unified Parkinson’s Disease Rating Scale part III; H&Y, Hoehn and Yahr; MoCA, Korean version of Montreal Cognitive Assessment; BAI, Beck Anxiety Inventory; BDI, Beck Depression Inventory; PFS, Parkinson’s Disease Fatigue Scale; SCOPA-AUT, Scales for Outcomes in Parkinson’s Disease-Autonomic Dysfunction; SD, standard deviation; IQR, interquartile ranges; OR, odds ratio; CI, confidence interval.
